# Inspiration from humans and microbes—and maybe a lot of luck: the “discovery” of *Shewanella oneidensis* MR-1 and extracellular electron transfer (EET)

**DOI:** 10.1128/jb.00242-24

**Published:** 2026-06-12

**Authors:** Kenneth Nealson

**Affiliations:** 1 Departments of Earth Science and Biological Sciences, University of Southern California 5116, Los Angeles, California, USA; Dartmouth College Geisel School of Medicine, Hanover, New Hampshire, USA

**Keywords:** *Shewanella*, extracellular electron transfer (EET), multiheme cytochrome (MHC)

## Abstract

In the early 1980s, it was generally believed that insoluble electron acceptors were unavailable to bacteria unless they could be solubilized and transferred from the cell exterior to the cell membrane for use in redox reactions. Thus, many potentially active oxidants, such as metal oxides, while thermodynamically capable of acting as electron acceptors for respiration, were thought to not be available to bacteria or archaea. However, in 1988, with the isolation and characterization of *Shewanella oneidensis* MR-1 (so-called because it was the first metal-reducing bug we found) (C. R. Myers, K. H. Nealson, Science 240:1319–1321, 1988, https://doi.org/10.1126/science.240.4857.1319), it became clear that microbial manganese oxide reduction was indeed possible by a process now known as extracellular electron transfer or EET. In this minireview, I first discuss the isolation and characterization of *S. oneidensis* MR-1 and its progression from a “must be verified” status to its position as a model organism for the study of EET. This is followed by a summary of the roles that *S. oneidensis* MR-1 played in opening many previously unknown windows of microbial metabolism.

## INTRODUCTION

Here, I will share the experiences I had while jumping into a new interdisciplinary field and asking a few simple questions, the answers to which have yielded a treasure trove of complex answers—and introduced me to a cadre of students, colleagues, and friends with whom I had the pleasure of sharing the experiences as we came to a few answers. One of the most exciting aspects of scientific research is the realization that something new has been learned — the so-called “aha moment.” Such moments are rare and wonderful, but they are almost always followed by a less enjoyable time: when the new finding does not sit well with one’s peers and/or with preconceptions in the field. Here, I deal with events that led to the search for an organism that uses insoluble metal (Mn^4+^ or Fe^3+^) oxides as electron acceptors for respiration: why we searched, how we searched, and how the story unfolded once we had an organism in hand.

The story begins in the 1970s with stimulation provided by discussions with aquatic geochemists who had detected very rapid rates of metal reduction that could not be explained by the chemistry of the environments where the reduction was occurring. Through these discussions, I became suspicious that an existing microbiological paradigm (i.e., that bacteria were not capable of respiration of insoluble compounds) just might be wrong. Almost 10 years later, my lab was treated to an “aha moment” with the isolation of bacteria that grew with insoluble manganese oxide (MnO_2_) as its only electron acceptor (1). This was followed by a slow progression of acceptance of the idea that *S. oneidensis* MR-1 (hereafter referred to as MR-1) was indeed capable of anaerobic respiration of metal oxides. Who would have imagined that MR-1 would become one of the model organisms for the study of microbial metal oxide reduction and other aspects of extracellular electron transfer (2–5)? Critical to the acceptance of this work were discussions with many colleagues (most of whom, with good reason, were politely skeptical!) who thought that there must be other explanations for our results.

To begin with, we needed to achieve consensus on the definition of anaerobic respiration, which we now take to be the flow of electrons to electron acceptors other than oxygen, thereby allowing the conversion of chemical energy to biologically useful energy in the form of ATP. As pointed out by Hunt et al. (6), the focus should be on electron flow, and not on the mechanism of ATP formation, as under some conditions, maintaining redox balance allows ATP synthesis not directly linked to the proton motive force. Two important developments aided progress in this endeavor: first, the development of a robust system for genetic analysis (7–10), and second, the publication of the MR-1 genome (11).

## LEARNING SOMETHING NEW

A common theme in research is the notion that new discoveries often occur when an “outsider” enters a new field. Different backgrounds and perspectives allow the newcomer to suggest things that the “old timers” might never consider. A good example of this is the discovery of bacteria that could oxidize methane anaerobically. In this case, the existing paradigm, which we were all taught, was that microbial methane oxidation was strictly an aerobic process: without molecular oxygen, methane oxidation was impossible. The outsiders were marine geochemists who reported methane removal in anoxic zones of marine waters and sediments (12, 13). In response to these reports, two groups of microbiologists tracked down the microbes responsible for the anaerobic methane oxidation, which are now called the ANME group of bacteria (14, 15). A similar experience was in store for me.

## IMPORTANCE OF INTERDISCIPLINARY DISCUSSION

In 1973, when I took my first job as an assistant professor of marine microbiology at Scripps Institution of Oceanography, the work of my laboratory was focused on the biochemistry, physiology, and ecology of luminous bacteria, with a particular interest in the regulatory mechanisms controlling the synthesis and activity of the enzymes involved in light emission (16), which I naively assumed would continue for the rest of my career. However, my laboratory was located next to the lab of a marine geochemist, Professor Ed Goldberg (12), who, while he conceded that my lab was doing good work, continued to challenge me to do “something important” (read—marine geochemistry!). Eventually, Professor Goldberg, along with Professors Gustav Arrhenius and Joris Gieskes (two other fine marine geochemists), convinced me to begin working on the role of bacteria in the oxidation and reduction of manganese. This was my first step into the world of geobiology, and the many unexplained facets of iron and manganese cycling in aquatic environments provided the impetus. Most of these mysteries had to do with reaction rates (usually much faster than the rates predicted by environmental geochemistry) and the potential role of bacteria in driving the redox cycle of manganese in aquatic environments.

Our studies began with the isolation of marine bacteria capable of oxidizing soluble Mn(II) to insoluble MnO_2_ (17, 18), and on the basis of this work, I was invited to join MAnganese NOdule Program (MANOP), an NSF-funded interdisciplinary and interlaboratory effort to study marine manganese nodules (I always suspected I was the “token biologist” that made our project interdisciplinary!). I began to educate myself in the areas of bacterial oxidation and reduction of iron and manganese (19, 20) and began our work searching for marine bacteria that could oxidize and/or reduce manganese. However, since deep-sea nodules grow very slowly (estimated to be on the order of a few mm/10^6^ years) (21) and are difficult and expensive to collect and study, our early work was focused primarily on the isolation and characterization of marine bacteria capable of oxidation of soluble Mn(II) to insoluble manganese oxides, and reduction of insoluble Mn(IV) oxides (e.g., MnO_2_) to soluble Mn(II).

## GOOD LUCK—FINDING THE RIGHT STUDY SITE

One of the scientists involved with MANOP was Professor Willard (Billy) Moore, an expert in isotopic dating of geological materials, with extensive experience working on manganese nodules in both marine and freshwater environments. Oneida Lake was well known to Billy for its abundant manganese nodules, and Billy introduced me to the studies that convinced me to look into this study site, beginning with the work of Walter Dean (22): studies that would be continued by many others, including my laboratory (23–29). With Billy’s urging, we began to study manganese cycling in Oneida Lake, NY, which proved to have many advantages over the deep-sea sites: (i) it was easily accessed by automobile and studied using small boats and SCUBA—no ocean-going ships needed; (ii) it has shallow shoals where oxidized MnO_2_ accumulates in the form of manganese nodules (22–24) and deeper sedimentary areas where the anaerobic sediments could be easily sampled (using SCUBA) to measure rates of MnO_2_ reduction; (iii) it is geologically very young, being formed after the last glaciation (about 12,000 years ago); (iv) it is part of New York Barge Canal system with its buoy markers allowing easy return to specific study sites (see Fig. 7); and (v) Cornell University has a Biological Station at Shackleton Point on the shore of Oneida Lake that we were graciously allowed to use.

## MORE GOOD LUCK

In 1985, I accepted a position as Distinguished Professor at the University of Wisconsin-Milwaukee’s Center for Great Lakes Studies, with labs located on the shores of Lake Michigan and only a 1-day drive to Oneida Lake. Several trips to Oneida Lake ensued, taking advantage of the use of the diving facilities and laboratories at the Cornell field station and Billy’s expertise. In Oneida Lake, we were able to do more observing, collecting, and measuring in a few days (and at whatever time intervals we needed) than could have been done in the deep sea in months. For example, using SCUBA, it was possible to set up flux chambers and measure in-situ metal reduction rates, something extremely difficult to do in the deep sea. Using these approaches, we soon had data supporting the rapid oxidation rates of Mn to insoluble Mn oxides (25). These studies and others that followed (27–29) yielded results that were in agreement with the previous geochemical studies of the lake (23–25). To my knowledge, Oneida Lake is one of the only lakes in the U.S. where rates of metal oxidation and reduction and whole-lake budgets of iron and/or manganese have been systematically documented.

## AN OUTSIDER IN OUR CAMP

At the onset of this work, I saw myself as the “old timer” touting the paradigm that I had been taught (19, 20), that while bacteria can catalyze the oxidation of manganese oxides (19, 20), the reduction of manganese oxide was unlikely to be directly linked to the respiratory metabolism of microbes (19, 20). But as the “outsider” Billy pointed out to me, Oneida Lake could only be explained by having very rapid rates of manganese oxide reduction under chemical conditions that were not consistent with the observed rates. Given these facts, I began to suspect that microbiological catalysis was the most reasonable explanation.

**Fig 1:**
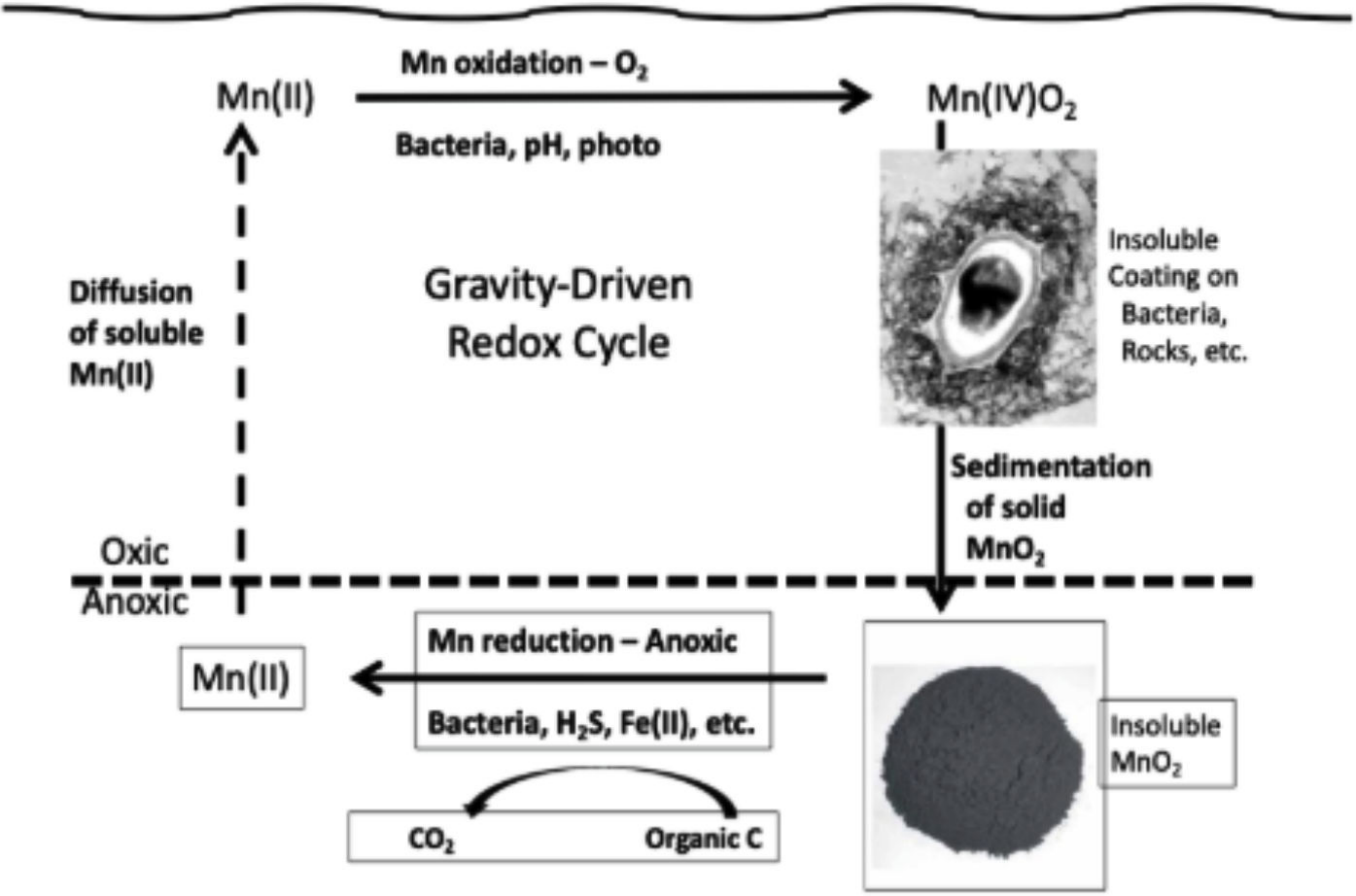
A generalized manganese cycle to explain the processes that occur in environments with redox boundaries (stratified lakes, and sediments). In oxic waters at and above neutral pH, Mn(II) is rapidly oxidized to Mn(IV) in the form of the insoluble oxide (MnO_2_) (31). Many aquatic bacteria (26) and phytoplankters (27) can catalyze the oxidation of Mn(II) to insoluble MnO_2_ that sinks to the sediments, along with sedimented organic matter. Manganese reduction occurs at the expense of organic carbon (28, 29) and the reduced manganese in the form of soluble Mn(II) that can diffuse into the overlying water to be oxidized again (28, 29).

As an aside, it was also fortuitous that we were working in freshwater with very low sulfate concentrations. In previous studies with marine bacteria, we had concluded that bacterial MnO_2_ respiration was a minor process if it occurred at all (29, 30). In anaerobic marine systems, high rates of sulfate reduction were seen, yielding high levels of hydrogen sulfide that abiotically rapidly reduced Mn(IV), leading to accumulation of soluble Mn(II) (30, 31). Thus, in marine sediments, respiration-linked Mn reduction is probably overwhelmed by sulfide production. In laboratory studies using sulfate-free seawater, we observed microbial manganese reduction, but it was driven by the excretion of metabolites that either acted as reductants and/or lowered the pH of the medium (30, 31). In no case were we able to show that manganese reduction was linked to the respiratory metabolism of the microbes until we began work in Oneida Lake, which provided a view of a whole-lake manganese cycle (Fig. 1).

In Oneida Lake, soluble reduced Mn(II) arrives in the spring of the year as runoff from the Adirondack mountains and is oxidized biologically and delivered via gravity to both deep and shallow zones. The manganese nodules (see Fig. 7) are found on these shallow shoals of the lake that remain oxic year-round and where oxidized sediments from the shoals are regularly swept into deeper waters by strong currents (24, 25).

## PROBLEM WITH RESPIRATORY METAL OXIDE REDUCTION

**Fig 2:**
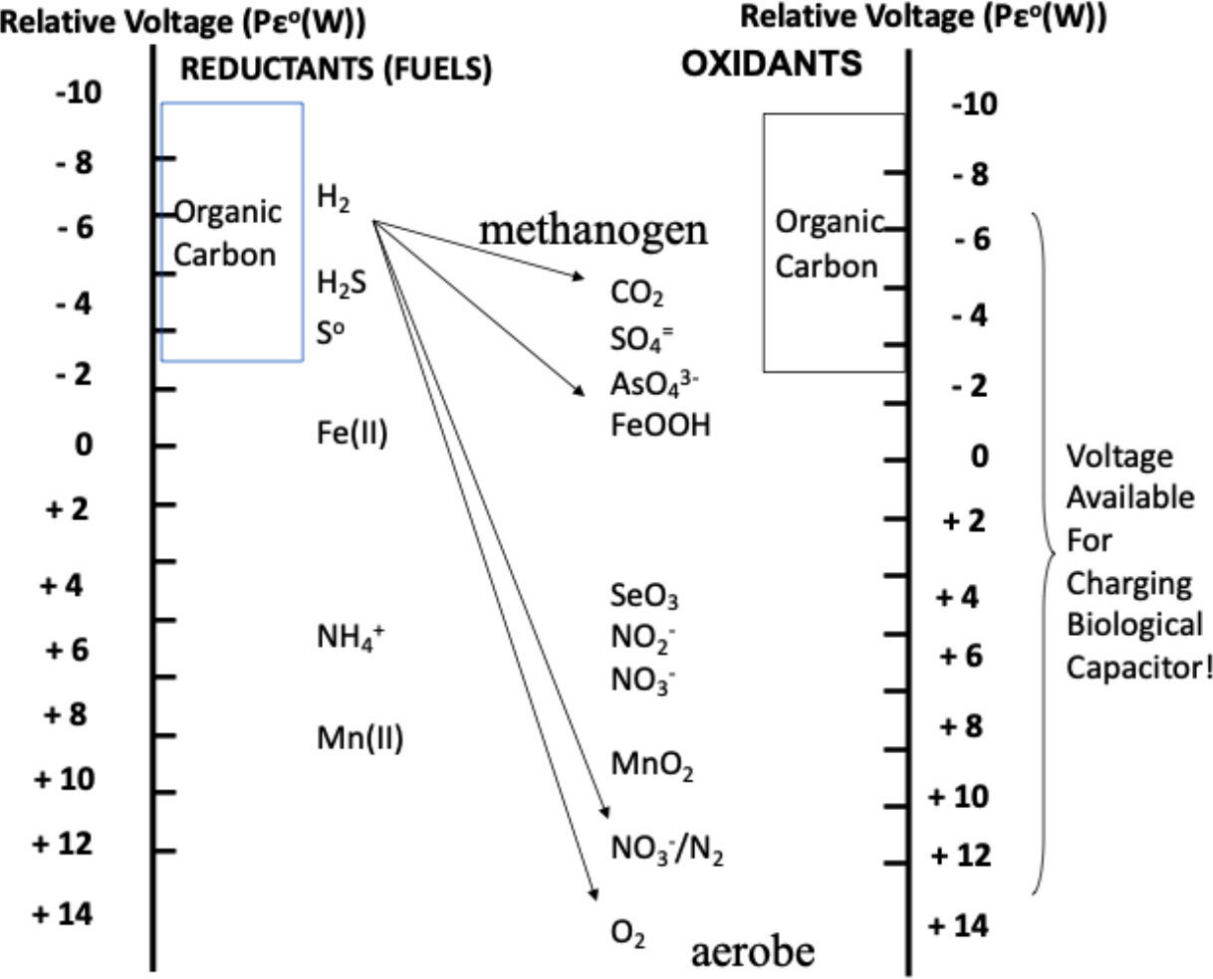
Electron flow and energy generation in the prokaryotic (bacterial plus archaeal) world. On the left side are some known electron donors, and on the right are electron acceptors. Relative voltages are expressed as Pe^o^(W) with the (W) indicating that the calculations are adjusted for reactions in water according to Stumm and Morgan (31). Arrows can then be used to connect any reductant to any oxidant, and if the slope is negative, there will be energy available, and some prokaryote will probably be known that can take advantage of this.

The reason that MnO_2_ was not thought to be a possible electron acceptor for bacteria was not based on its redox properties, but on the fact that it is insoluble. Solubilization requires MnO_2_ to be chemically reduced to Mn(II), rendering it unacceptable as an electron acceptor based on our knowledge at the time. The redox potentials of various MnO_2_ minerals are in the same range as that of nitrate (31), so thermodynamically, Mn(IV) should be a good candidate for anaerobic respiration if it could be transported into the inner membrane where the electric potential is converted to a chemo-osmotic gradient (called the proton motive force or PMF) that can be used by the bacteria to convert ADP to ATP and to drive motility membrane transport (Fig. 2).

However, the crux of the problem is that MnO_2_ is a solid and should not be able to interact with the electron flow mechanism on the inner membrane of the cell. All known manganese oxides are solids, and even small crystals are hundreds of microbial body lengths in diameter. How could a microbe possibly use these gargantuan electron acceptors? One solution to this apparent dilemma at first glance seems rather simple; if the electron acceptor cannot be moved to the inner cell membrane, why not move the electrons to the outside of the cell? This would allow the cells to generate a PMF via electron flow in the usual way, coupling standard electron transport to an electron transfer component to the cell exterior (i.e., the cells would just need to evolve a conductive element that could move electrons from the inner membrane to the cell exterior and make sure that they are delivered to the correct place). However, no such thing was known, and there was a strong prejudice against it (19, 20).

Why the prejudice? First, because no one had observed such activity (although it is not clear that anyone had looked for it!), and second, because moving electrons to the cell exterior would require the “invention” (i.e., evolution) of many new things, including electron transfer components, trans-membrane electron carriers, porins, and outer membrane electron transfer components, to name a few. The bottom line is that this is NOT something simple: it would require new types of electron carriers with new abilities distinctly different from the cytochromes that were known at the time. Thus, the challenge was to find an organism that was capable of metal oxide respiration and have a look at it. We opined that if such microbes existed, Oneida Lake should be a very good place to find them.

## SEARCHING FOR THE MICROBIAL CATALYSTS

**Fig 3:**
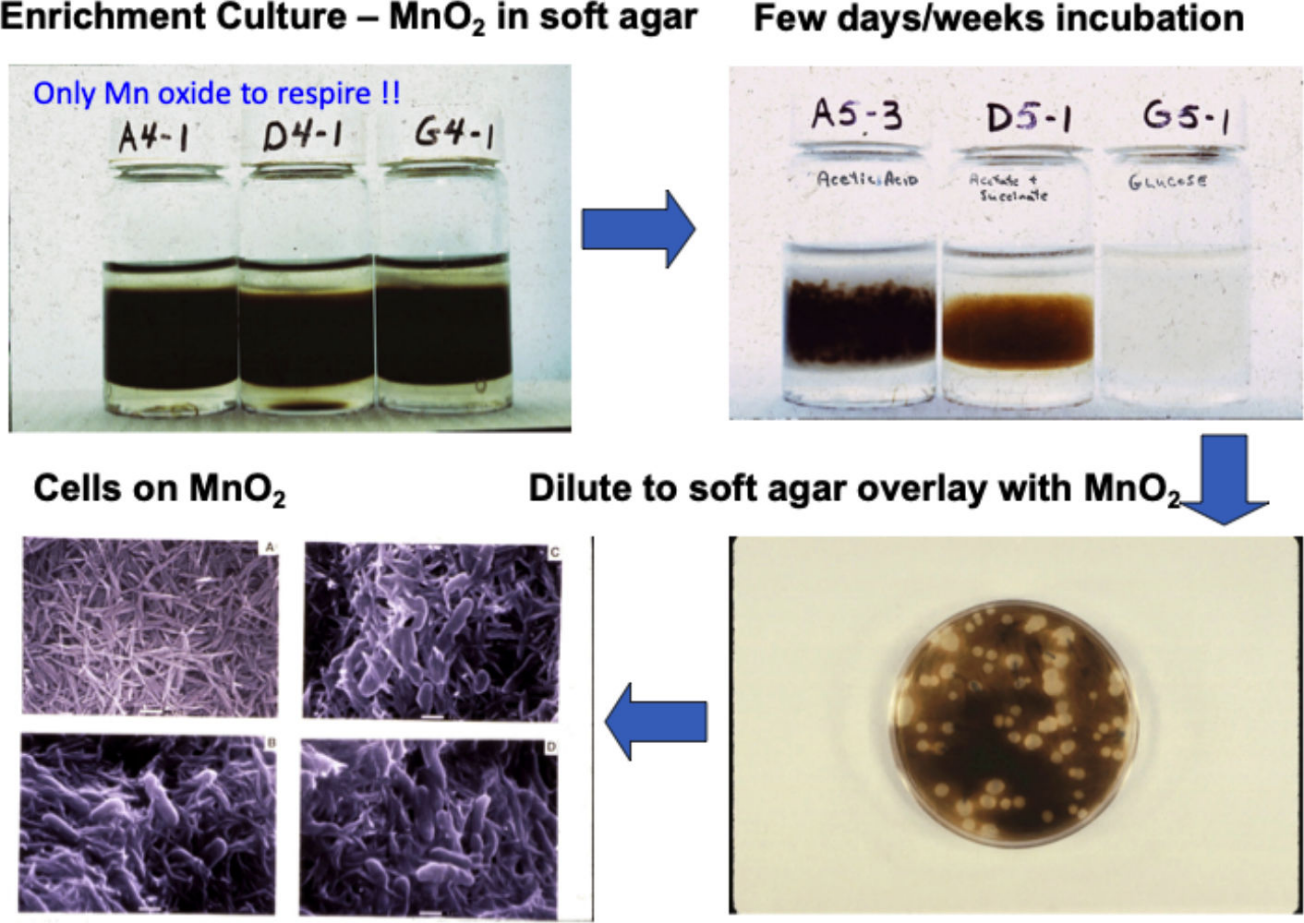
Enrichment vials containing MnO_2_ as the sole electron acceptor. Upper left: vials at T = 0. Upper right: vials after 5 days of incubation (A = acetate, D = lactate, G series = glucose). Lower right: dilution plating of *S. oneidensis* MR-1 spread on agar plate that is overlain with soft agar containing MnO_2_ and lactate and incubated anaerobically. The lower left panel shows *S. oneidensis* MR-1 cells attached to MnO_2_ particles. This method has also been used for estimations of manganese reduction in the field (33).

Previous studies of Oneida Lake clearly indicated that it was a dynamic system with literally tons of Mn being rapidly recycled via oxidation of Mn(II) and reduction of MnO_2_ (26–29), with the rates of both processes occurring orders of magnitude faster than those predicted given the chemistry of the water or sediments of the lake. Thus, our goal, after being convinced by the measured rates, was to find organisms that could catalyze the MnO_2_ reduction and perhaps account for the observed rapid rates.To do this, we used a classical and simple approach, called enrichment culturing, using sediment samples from the deep zones of Oneida Lake as the inoculant and providing these samples with various electron donors (e.g., glucose, lactate, and acetate) but only manganese oxide (MnO_2_) as the electron acceptor. The enrichments were done in tightly capped 15 mL vials containing water and/or sediments from Oneida Lake and finely ground MnO_2_ particles that were suspended in soft agar (0.75%), which kept the metal oxide particles in suspension while allowing the bacteria to swim freely via flagellar motility (Fig. 3). After inoculation, a layer of mineral oil was added to slow the rate of oxygen diffusion into the enrichments. The aerobes and facultative anaerobes quickly used up any oxygen that was present, yielding simple and effective anaerobic enrichments for microbes using other electron acceptors. As a side note, this approach has also served as a good method for teaching, as the active zones of metal reduction can easily be seen as zones of clearing with the disappearance of the black metal oxide, and samples can be taken with syringes for both observation and organism isolation (Fig. 3). Such enrichments have also been used in environmental studies to identify areas of active metal reduction in natural waters, especially in anaerobic interfaces in stratified water bodies (32).

Single colonies were isolated by dilution plating onto 1.5% agar medium that was then overlain with the same soft agar medium (containing Mn oxide) that was used for the enrichments, and plates were incubated in an anaerobic bag or chamber (Fig. 3). The assumption was that any colonies that grew must be using the manganese oxide as an electron acceptor. These observations, first made by Dr. Charles Myers (1) in my laboratory, provided the “aha moment”—a moment never forgotten! Somehow, this respiratory organism was growing without oxygen or any other known electron acceptor except insoluble MnO_2_ on a non-fermentable energy source: imagine our surprise and delight!

Reinoculation of fresh enrichment vials using lactate as the energy source resulted in rapid metal reduction visible in hours or days (Fig. 3). Note here that the original enrichments were not pure cultures, and that even acetate vials showed some manganese oxide reduction, albeit at much slower rates and never as pure cultures. Microscopic analyses of the enrichments revealed both high numbers and a high diversity of microbes. With molecular genetics in mind for the future, we chose to look for a fast-growing, oxygen-tolerant organism that upon transfer to an anaerobic environment could grow with MnO_2_ as its only electron acceptor. After a few weeks of culturing, several likely candidates were found, some of which, using standard taxonomic analyses, fell into the group, called *Alteromonas putrefaciens*, and one was selected and named MR-1 to indicate it was the first metal-reducing bug we had found (1). With the development of molecular taxonomic methods, strain MR-1 was moved to the genus *Shewanella* that had been recently proposed to include several strains formerly identified as *Vibrio* and *Alteromonas* (32, 34), and our isolate was eventually named *Shewanella oneidensis* MR-1 (35).

Initial experiments with *S. oneidensis* MR-1 were aimed at gaining insight into the mechanisms of MnO_2_ reduction and how it was impacted by pH, temperature, the ratio of cells to MnO_2_, the impact of the presence of other electron acceptors (oxygen, nitrate, etc.), the importance of cell contact for efficient metal reduction, and the impact of metabolic poisons on the rates of metal reduction. Among the findings were that the reduction of MnO_2_ proceeded well at circumneutral pH (6 to 8), was inhibited by temperatures below 4°C and above 35°C, and was inhibited by several antibiotics and poisons. Rates of reduction were proportional to bacterial abundance, while increasing the amount of oxide had little impact on the rate of reduction at a constant bacterial concentration, and oxygen was a strong inhibitor of MnO_2_ reduction, and the cells grew well aerobically (1). When the bacteria were separated from the MnO_2_ via a dialysis membrane, the rate of reduction was much slower than when the bacteria had direct access to the MnO_2_ particles (Myers and Nealson, unpublished).

## THE TABLES HAVE TURNED

With these results in hand, we had convinced ourselves that strain MR-1 was capable of anaerobic respiration of metal oxides, but given the existing paradigm, it was not so easy to convince others. So now, I was now the “outsider” arguing that metal oxide respiration was real. But, as luck would have it, a similar report of another pure culture of metal-oxide reducing bacteria (eventually placed in a new genus called *Geobacter*) appeared simultaneously (36) and proved to be a great help in convincing the nay-sayers that anaerobic respiration of metal oxides was indeed real.

In the ensuing 38 years, a lot (actually a huge amount!) has been learned about *Shewanella*, which has become a model organism for the study of metal oxide reduction, with hundreds of species and strains being isolated from all around the planet and more than 50 different genomes being sequenced (2–5). *Shewanella* species have been found in marine, freshwater, and sedimentary environments around the world—they are probably one of the most cosmopolitan groups of microbes known, being especially competitive in any environment that has fluctuating redox zones. These details will not be discussed here, but I recommend several reviews (2–5). My message is focused on the initial isolation of *S. oneidensis* MR-1, its ability to catalyze metal oxide reduction via extracellular electron transfer, the growth of knowledge and appreciation of EET, and what this has brought to the field of microbiology.

## HOW DO THEY DO IT?

Early progress pertaining to the mechanism(s) of EET involved both molecular/genetic and biochemical studies to identify the genes and proteins that catalyzed EET. The *Shewanella* work in my lab was led by two outstanding postdocs, Charles Myers and Daad Saffarini. In 1992, Myers and Myers (37) reported that MR-1 possessed abundant c-type cytochromes when grown anaerobically, and that more than 80% of the bound cytochromes were localized to the outer membrane. In 1993, genetic analyses revealed several EET-negative mutants, the characterization of which led to the identification in MR-1 of both regulatory (*etrA*) and structural (*mtr*A and *mtr*B) genes that were required for EET (7–9, 38). The sequence of *cymA*, which coded for a periplasmic tetraheme cytochrome (also needed for EET), added to the excitement (38). MtrA was found to be a decaheme cytochrome located on the outer membrane, while MtrB was a porin-like protein also bound to the outer cell membrane of MR-1. Both were directly involved in metal reduction.

At this point, a major event occurred—the formation of the “Shewanella Federation” (SF), a DOE-funded program overseen by Ari Petrinos and Dan Drell. It was headed by Jim Fredrickson from the Pacific Northwest Laboratory and myself and involved about 20 research teams that were recruited from many different universities to join the MR-1 research effort. The SF met regularly to discuss progress and plan further experiments to broaden the collective knowledge. I suspect that 100s of publications can be traced back to SF activities, and much of what we now know about metal reduction and EET can be attributed to the support (both financially and scientifically) of the DOE, which was also responsible for funding the genome sequencing project that led to the publication of the MR-1 genome (11). It was becoming clear that MR-1 and EET were important things to understand, and at Environmental Molecular Sciences Laboratory (EMSL), EET was chosen as a “Grand Challenge,” with Jim Fredrickson and John Zachara leading this effort. Many of the SF labs joined in this effort, and one by one, it became clear that things were far more complex and interesting than any of us had imagined (39–43).

**Fig 4:**
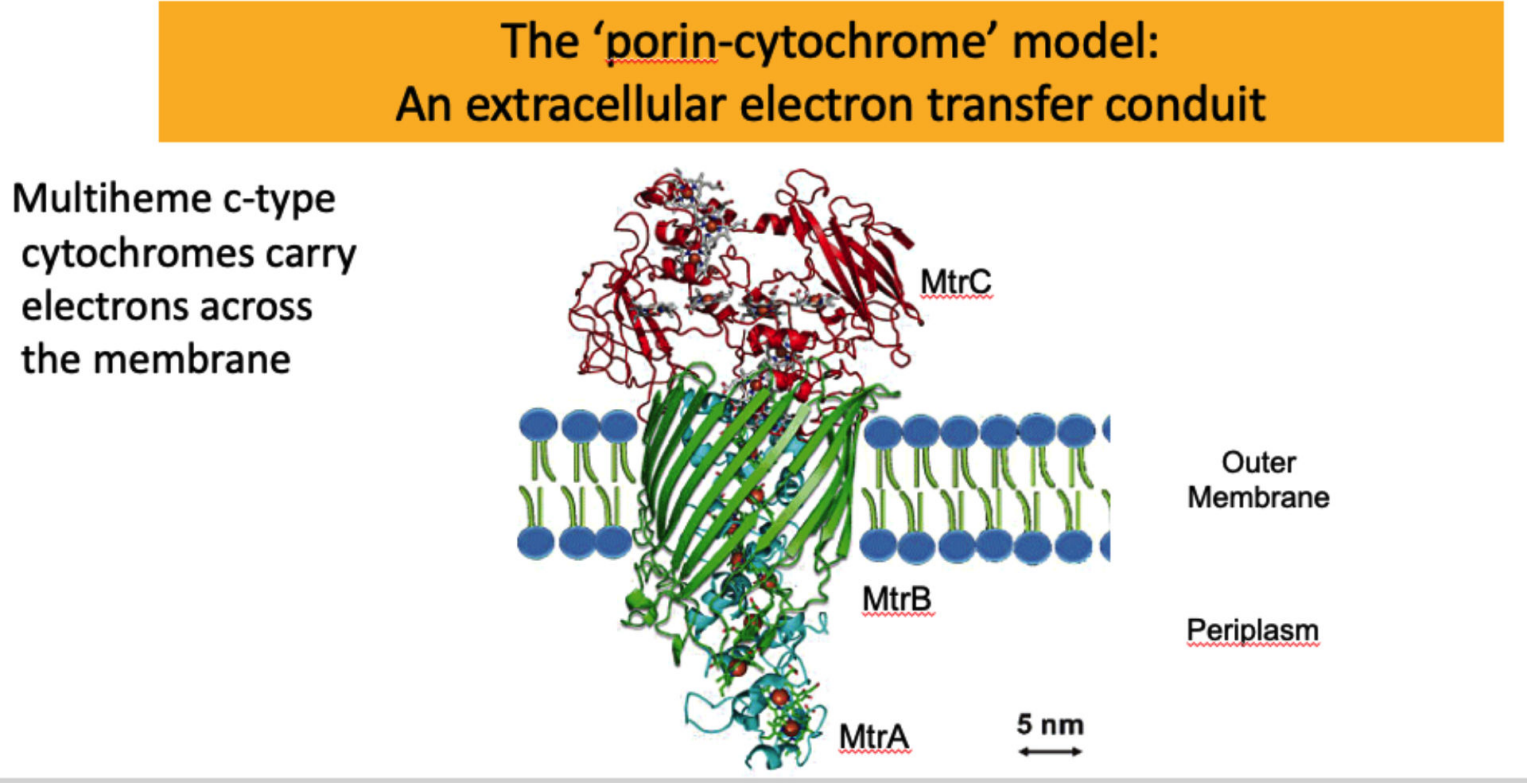
Porin/cytochrome model of electron transfer to the exterior (EET) (44). MtrA and MtrC move electrons from the periplasm through the pore provided by MtrB, allowing respiratory EET to insoluble substrates outside the outer membrane. Red dots indicate the iron in heme groups that are located to allow electron hopping (44–47). Figure courtesy of D. Richardson.

But, the original goal of my laboratory, which was to explain the mechanism of metal oxide reduction by MR-1 and understand the ecophysiology of this process, remained elusive. That is: how does MR-1 move electrons from the inner membrane to the cell exterior, and how is that movement controlled? To my delight, the solution to this question was put to rest via a collaborative effort with the group of Richardson at the University of East Anglia in the UK in collaboration with EMSL scientists—it was called the porin-cytochrome model, as shown in Fig. 4 (44–47), in which EET in MR-1 could be explained by CymA carrying the electron to MtrA (transmembrane multiheme cytochrome) through the outer membrane (protected by a porin called MtrB) to MtrC (outer membrane multiheme cytochrome), which could then transfer the electrons to other multiheme cytochromes on the outer membrane (e.g., OmcA). Virtually, all the cytochromes involved in this process were multiheme (either tetraheme or decaheme) cytochromes (MHCs) working together to move the electrons to the cell exterior (44–47). It seemed we were near to understanding the EET situation, but there was still more to learn.

## OTHER VARIATIONS

In 2000, it had been proposed that extracellular electron shuttles, such as quinones and phenazines, produced endogenously (48, 49) or added exogenously could be involved in EET, being reduced by the cell and diffusing to insoluble electron acceptors. This was followed by reports by Marsili et al. (50) and Edel et al. (51) that MR-1 secretes riboflavin that, when reduced, can act as an electron shuttle for long-distance EET. At about the same time, Byong Hong Kim (who would later be a sabbatical visitor in my lab) reported that MR-1 was capable of direct reduction of graphite electrodes (52, 53). These reports invigorated the community, getting many others, including my lab group, into the study of what later grew into the field of electromicrobiology (54–56).

In 2006, Gorby et al. (57) reported the production of bacterial nanowires by MR-1. These structures turned out to be membrane extensions of distances up to several nanometers that allowed for long-distance direct electron transfer (EET) to distant electron acceptors. This report stimulated a lot of further work describing the biophysical properties of the membrane extensions of MR-1 (58, 59), which are distinctly different from the nanowires produced by *Geobacter* species now known to be stacked polymers of tetraheme cytochromes (60–63).

**Fig 5:**
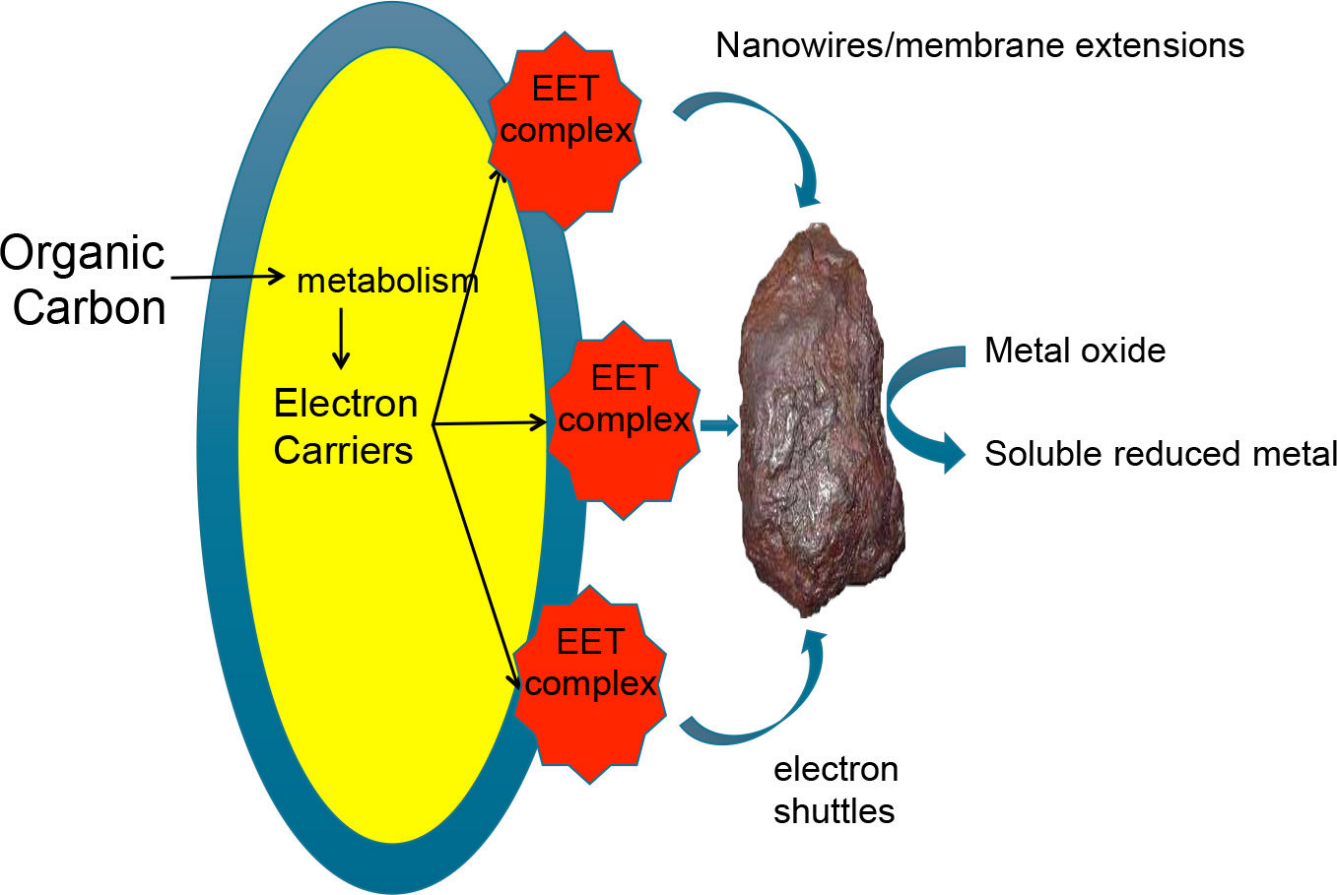
Pathways of EET. The importance of the EET complex (porin-cytochrome system) is clear, as shown here. Once the electrons have moved to the cell exterior, they can be used directly by the outer membrane cytochromes or by nanowires (membrane extensions containing the extracellular cytochromes). In addition, the EET complex can reduce soluble electron carriers (either endogenous or exogenous) to carry electrons to the insoluble metal oxides by diffusion.

With these variations in hand, EET was suddenly a far more abundant and exciting area of study, as outlined in Fig. 5 below.

But, more variations were yet to come. EET-capable microbes could also transfer electrons to other bacteria via a process called direct intercellular electron transfer (DIET) (64). This report opened a new world of microbial interactions in which some microbial cells could act as electron donors and others as electron acceptors—providing a pathway to microbe-microbe syntrophy via electron transfer. Since that time, many examples have arisen in which DIET-like electron transfers have been seen that support unexpected modes of bacterial syntrophic interactions (65–67).

**Fig 6:**
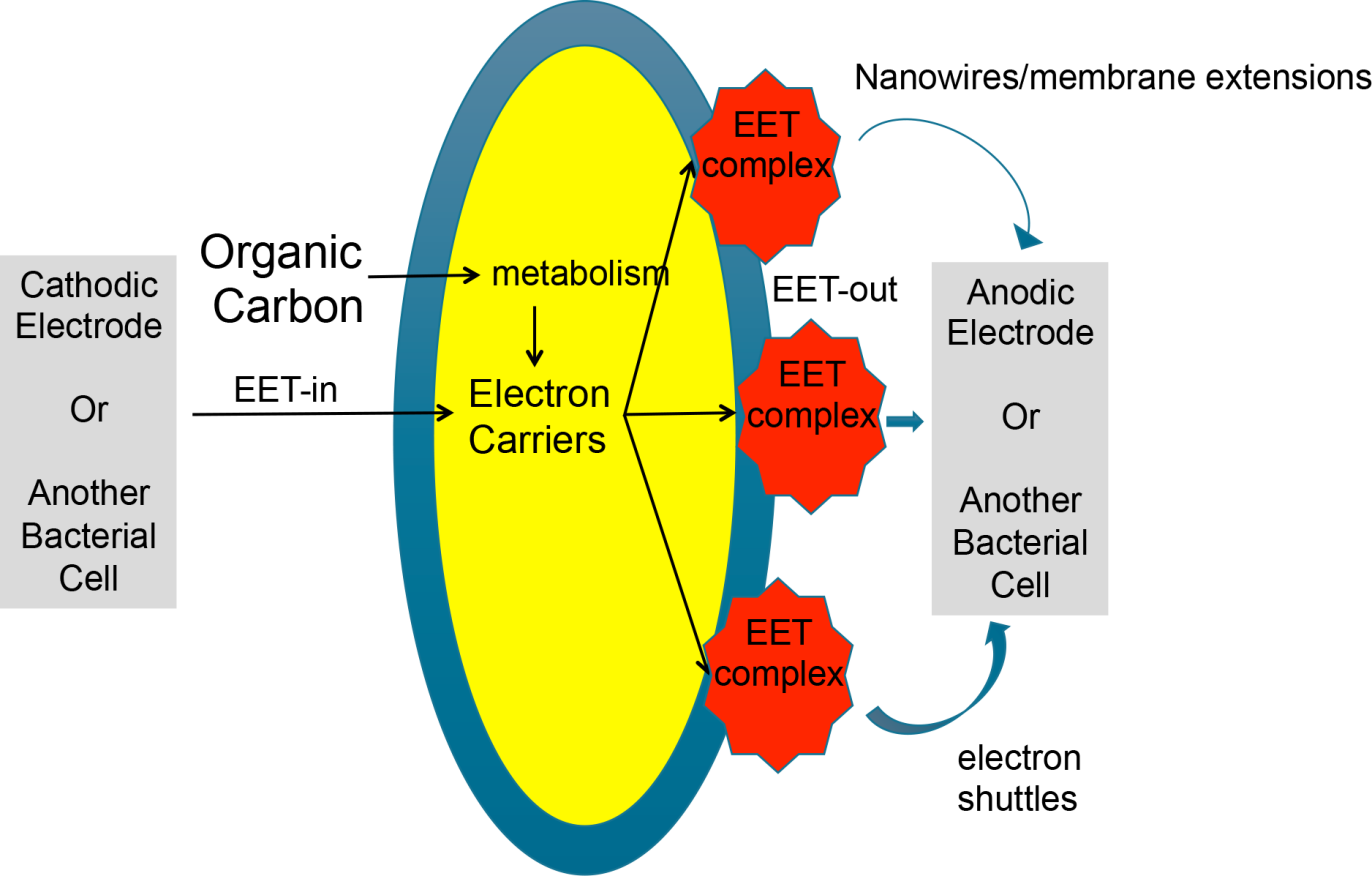
Versatility and variability of EET. Organic electron donors are transported into the cell, and electrons are moved to electron carriers. Electrons are carried to the EET complex to the outer membrane where they are used to reduce solid metal oxides (allowing electron flow and energy production inside the cell) by direct EET-out. Electrons can also enter the cell from cathodic electrodes or other cells via EET-in, the mechanisms of which are under study (69–72). These electrons can be moved to metal oxides by direct EET-out via indirect (shuttle-mediated) EET-out. The nature and detailed mechanisms of nanowires and membrane extensions, both of which allow for EET at a distance, are under study in many laboratories.

Implicit in the discussion of syntrophic EET is the notion that some cells are capable of the uptake of electrons, thus allowing energy transfer between cells. These thoughts led several laboratories to demonstrate that cathodic electrodes could be used to enrich for electron-utilizing bacteria (68–71). Rowe et al. (72) defined a pathway for the uptake of electrons in MR-1, and many laboratories have made it clear that many different bacteria are capable of both EET-in as well as EET-out, including many chemolithotrophs. In MR-1, we showed that reversing EET is not just a simple biocatalysis driven by the known cytochrome players, but that electrons flowing into the cells could be coupled to energy conservation. This finding, once challenged, has been confirmed by other groups, and novel genes have been shown to be involved in this process (72), begging the question of whether bi-directional electron flow gives MR-1 an ecological advantage in the redox-variable environments in which it thrives. Fig. 6 presents a summary of the above discussion—it is a picture of microbial metabolism that could not have been imagined 30 years ago.

## THE IMPORTANCE OF MULTIHEME CYTOCHROMES (MHCS)

In nearly all examples of EET that have been described, one thing seems constant: the importance of MHCs in EET, whether inward or outward. What at first appeared to be a rare occurrence has turned out to be a widespread ability; an evolutionary “invention” that has arisen many times using a wide range of proteins to accomplish similar jobs. It now seems clear that EET is not at all an isolated or unusual phenomenon, and that MHCs, rather than being rare proteins, are found in many groups of microbes (2, 3, 12, 13, 35, 73–76), many of which have not yet been shown to be EET-capable. I suspect that what has been discovered up to now is the “tip of the iceberg,” and that MR-1 has helped convince us to look “below the surface” in search of an expected wide variety of interactions powered by electron exchange. It seems inescapable that electron flow-driven similar syntrophic interactions will be found to be very common occurrences. To this end, Garber et al. suggested that MHCs are far more widely distributed than suspected, being common in *Vibrio*, *Aeromonas*, various archaea, and in gram-positive species, with a wide variety of MHCs being found in many different microbes (73–76).

## WHERE ARE WE GOING?

Looking to the future, it is now apparent that EET is something far more important than the ability to reduce insoluble metal oxides or to power microbial fuel cells. One should expect to find many more genera using MHCs for survival and growth via syntrophic interactions. If you can imagine almost any type of metabolism (especially anaerobic metabolism), a syntrophic interaction driven by EET and enabled by MHCs will likely be found to make it happen. Many laboratories are working on EET-capable microbes, and new insights seem to arrive monthly, especially with the advent of new and better methods of generating and identifying genes involved in EET. As for the future, I expect that many more electrochemical syntrophic interactions will be found, and do not be surprised if these electrotrophs are found to be “talking to” algae, fungi, and even animals—electron flow is undoubtedly a universal language. In Fig. 5 and 6, it is easy to see that EET (both in and out) offers so many advantages for bacteria and archaea to interact and accomplish metabolic tricks that were previously thought to be impossible, such as anaerobic methane oxidation (12, 13). While more than 38 years of research enjoyment has been supplied by *S. oneidensis* MR-1, the importance of MR-1 and the many other EET-capable microbes lies both in the mechanistic details that have been elucidated and the realization that similar mechanisms are widely distributed and may well add many new chapters to the continuing story of microbial eco-physiology.

As a final note, I must remind us all that this huge amount of information was obtained almost totally from the study of two bacterial genera (*Shewanella* and *Geobacter*). In our original enrichments from Oneida Lake, we saw many other organisms in the primary enrichments. We selected for oxygen-tolerant, fast-growing bugs and ended up with *S. oneidensis* MR-1. If I were 30 years younger, I would return to Oneida Lake and have another look. Almost certainly, there are interesting microbes with new stories to tell. Think about it.

## CONCLUSION

(i) The search for microbes capable of respiration (dissimilatory reduction) of metal oxides was driven by discussions with geochemists who knew of places where very rapid rates of metal reductions were occurring (e.g., Oneida Lake, NY). New ideas and inspiration often come in the form of interdisciplinary discussion.

(ii) The sediments of Oneida Lake yielded many metal-oxide reducing bacteria, including *S. oneidensis* MR-1. Finding the right study site is often the difference between success and failure—in this case, it was a stroke of luck meeting Billy Moore and “discovering” Oneida Lake.

(iii) Like many other discoveries, once the “aha moment” had occurred and had been confirmed, the field moved forward: in this case, revealing that EET is a common metabolism-enabling process with a variety of mechanisms involved, all of which involve electron flow. One of the most exciting things in science is the realization that something new has been found, but convincing others it is true often involves a decade (or more) of work. Inspiration can come from unexpected directions. MR-1 itself became a source of inspiration as the breadth of its metabolic abilities was unveiled—hundreds of laboratories around the world have joined in the intellectual fun.

## SOME NECESSARY COMMENTS

**Fig 7:**
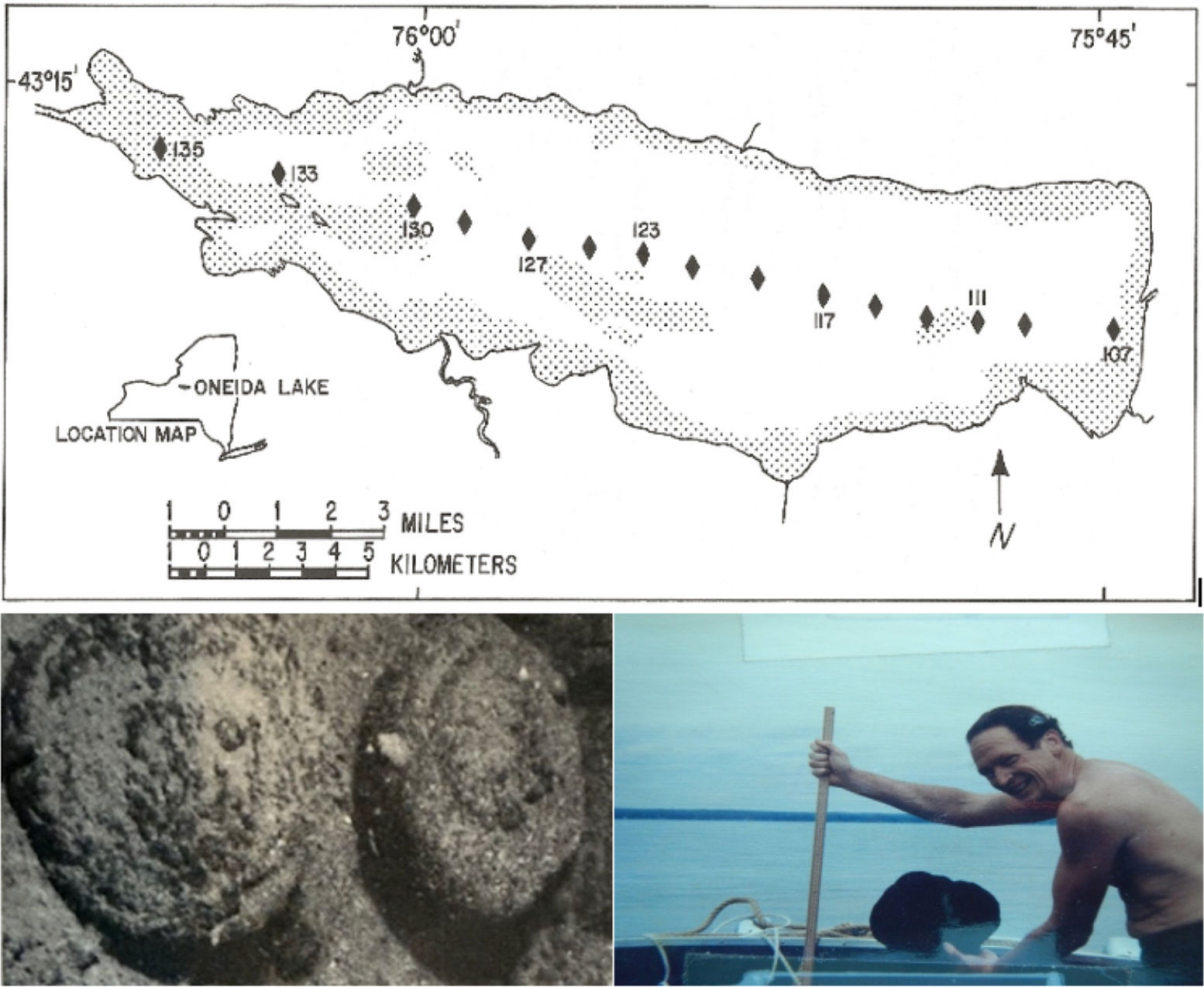
Top: map of Oneida Lake. Black diamonds show the location of buoys used by us for site location and *in situ* experiments. Shaded areas denote shoal areas 3–5 m in depth. Bottom left: manganese nodules on lake floor (each nodule is approximately 6–8 in in diameter). Bottom right: manganese nodule aboard research vessel.

First, as is always the case, nothing I have described or discussed in this review was done by me alone: (i) the ideas came from discussions with colleagues, students, and postdocs; (ii) the work was done by graduate students, postdocs, and colleagues; and (iii) the funding came from many different sources (DOE, ONR, AFOSR, and NSF). Second, here, I have focused almost entirely on *S. oneidensis* MR-1 and the pathway from discovery to prediction. *Geobacter* has its own story, and there will be many others. Third, a message for the young at heart is simply that you should not be discouraged when someone says, “It can’t be so!”—you may be onto something very interesting and new. Science is meant to change with time, driven by new ideas, new technology, and new methods that allow previously unavailable resolution that enables new and different interpretations. I fully embrace these unexpected changes and revel in the small part I have had the opportunity and privilege to participate in this journey of discovery and knowledge. It makes one smile on the way to work—how lucky I have been. As a final point (and a request from some reviewers), I present Fig. 7, a view of Oneida Lake and some of our workers—this is where it all began.
